# High-Quality NMR Structure of Human Anti-Apoptotic Protein Domain Mcl-1(171-327) for Cancer Drug Design

**DOI:** 10.1371/journal.pone.0096521

**Published:** 2014-05-02

**Authors:** Gaohua Liu, Leszek Poppe, Ken Aoki, Harvey Yamane, Jeffrey Lewis, Thomas Szyperski

**Affiliations:** 1 Department of Chemistry, State University of New York at Buffalo, Buffalo, New York, United States of America; 2 Molecular Structure, Amgen, Thousand Oaks, California, United States of America; 3 Protein Science, Amgen, Thousand Oaks, California, United States of America; National Institute for Medical Research, Medical Research Council, London, United Kingdom

## Abstract

A high-quality NMR solution structure is presented for protein hMcl-1(171–327) which comprises residues 171–327 of the human anti-apoptotic protein Mcl-1 (hMcl-1). Since this construct contains the three Bcl-2 homology (BH) sequence motifs which participate in forming a binding site for inhibitors of hMcl-1, it is deemed to be crucial for structure-based design of novel anti-cancer drugs blocking the Mcl1 related anti-apoptotic pathway. While the coordinates of an NMR solution structure for a corresponding construct of the mouse homologue (mMcl-1) are publicly available, our structure is the first atomic resolution structure reported for the ‘apo form’ of the human protein. Comparison of the two structures reveals that hMcl-1(171–327) exhibits a somewhat wider ligand/inhibitor binding groove as well as a different charge distribution within the BH3 binding groove. These findings strongly suggest that the availability of the human structure is of critical importance to support future design of cancer drugs.

## Introduction

The malfunctioning of cellular apoptosis [1] is a major hallmark of cancer. The regulation of apoptosis depends on the family of Bcl-2 proteins which contain one or several Bcl-2 homology (BH) sequence motifs. Based on their function and the similarity of their respective BH sequence motifs, these proteins can be grouped into three classes [2],[3]: (i) multi-domain pro-apoptotic proteins such as Bax and Bak, (ii) anti-apoptotic (i.e., pro-survival) proteins such as Mcl-1, Bcl-1, Bcl-x_L_, Bcl-w and Bfl-1/A1, all of which exhibit a similar architecture as Bax and Bak, and (iii) several pro-apoptotic proteins comprising only a single BH3 sequence motif such as Bid, Bad, Bim, Puma, Noxa, Hrk, Bmf, and Nbk/Bik (‘BH3-only’ proteins). The BH3 motif of class (iii) proteins forms an amphipathic α-helix which interacts specifically with a hydrophobic pocket formed in both pro-apoptotic class (i), and anti-apoptotic class (ii) proteins with participation of their respective BH motifs [2],[3]. Inhibition of the resulting protein-protein complex formation offers a promising strategy to treat cancer. For example, the small molecule Bcl-2 antagonist ABT-737 [4] inhibits anti-apoptotic class (ii) proteins Bcl-x_L_, Bcl-w and Bcl-1, and a congener [5] that can be orally administered is currently in clinical trials.

The anti-apoptotic, pro-survival 350-residue protein Mcl-1 (‘myeloid cell leukemia-1’) [2] is primarily anchored in the outer mitochondrial membrane by a C-terminal trans-membrane domain and contains three BH sequence domains: BH3 (residues 209–223), BH1 (252–272) and BH2 (residues 304–319) [2]. Mcl-1 inhibits death receptor-induced apoptosis by selectively binding to truncated Bid (tBid) [6] and can sequester endogenous Bak to block Bak-mediated cell death. Moreover, Mcl-1 interacts with several BH3-only proteins (Bim, Bid and Puma, Noxa and Bak). Hence, Mcl-1 plays an early role in response to signals directing either cell survival or cell death [2] and has been shown to be up-regulated in numerous malignant tumors. Approaches abrogating the Mcl-1’s anti-aptototic function either by reducing its abundance or by inactivating its functional BH3-binding groove show great promise for the cancer treatment [2],[4],[6],[7]. Here we present the high-quality NMR solution structure of polypeptide segment 171–327 of human Mcl-1 (hMcl-1) which comprises the three BH motifs deemed to be crucial for structure based drug design.

## Results and Discussion

**Figure 1:**
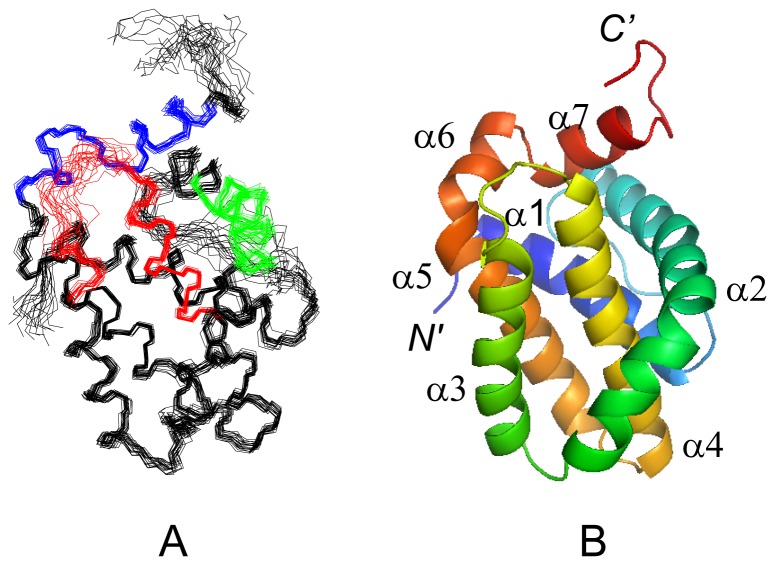
NMR structure of hMcl-1(171–327). (**A**) Backbone of the 20 CYANA conformers representing the solution structure of hMcl-1(171–327) after superposition of backbone N, C^α^ and C’ atoms of the α-helices for minimal rmsd. The three BH sequence motifs are colored in green (BH3), red (BH1) and blue (BH2), respectively. (**B**) Ribbon drawing of the lowest energy conformer of hMcl-1(171–327). α-helices α1-α7 are labeled and colored differently, and the N- and C-termini are labeled as “*N’* ” and “*C’* ”. The figures were generated using the programs MOLMOL [36] and PYMOL [37].

**Figure 2:**
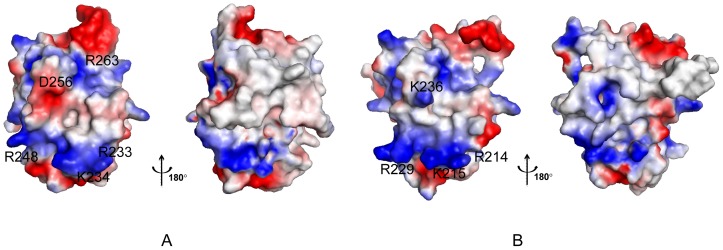
Electrostatic surface potentials. (**A**) For human hMcl-1(171–327) in the orientation shown in Figure 1 (left) and after rotation by 180° about the vertical axis (right). Surface colors (blue for positively charged; red for negatively charged) indicated the electrostatic potential calculated by using PYMOL [37] and its default vacuum electrostatics protocol. (**B**) Same as in (A) but for mouse mMcl-1(152–308).

A high-quality NMR structure of hMcl-1(171–327) was obtained (Table 1) and the coordinates were deposited in the PDB [8] (accession code 2mhs). The structure comprises seven α-helices α1-α7 (residues 173–191, 204–235, 240–253, 262–280, 284–301, 303–308 and 311–319) arranged to form the characteristic ‘Bcl-2 core’ structure [9] (Figure 1). The helices are locally and globally well-defined, while the C-terminus (residues 320–327) and the loops connecting, respectively, helices α1 and α2, helices α3 and α4, and helices α4 and α5 are flexibly disordered. The central helix α4 is surrounded by the other six helices, with α1, α2, α3 and α5 packed around one side, and α6 and α7 packed against its N-terminus. Helices α2, α3, α4 and α7 participate in forming the BH3 binding groove. The electrostatic protein surface potential is positive at both ends of the BH3 binding groove (due to the presence of Arg 233, Lys 234, Arg 248 and Arg 263) and negative at the side of helix α3 side (due to Asp 256) (Figure 2). This shows that the charge distribution in the BH3 binding groove of hMcl-1(171–327) differs distinctly from other anti-apoptotic proteins [10].

**Table 1:** Statistics of hMcl-1(171–327) NMR Structure.

Completeness of stereo-specific assignments [%]^a^	
^α^CH2 of Gly	55 (6/11)
^β^CH2	38 (27/71)
Val and Leu methyl groups	100 (27/27)
Conformationally restricting distance constraints	
Intra-residue [i = j]	1052
Sequential [|i-j| = 1]	1062
Medium range [1 < |i-j| < 5]	1197
Long range [|i-j| ≥ 5]	1058
Total	4369
Didedral angle constraints	
φ	113
ψ	113
Number of constraints per residue (170–327)	29.1
Number of long range constraints per residue (170–327)	6.7
CYANA target function [Å^2^]	0.88±0.12
Number of distance constraints violations per CYANA conformer	
0.2–0.5 Å	0
> 0.5 Å	0
Number of dihedral-angle constraint violations per CYANA conformer	
> 5^°^	0
Average rmsd to the mean CNS coordinates [Å]	
Α-helices,^b^ backbone heavy atoms N, C^α^, C’	0.42±0.05
Α-helices,^b^ all heavy atoms	0.88±0.07
Residues 172–312, backbone heavy atoms N, C^α^, C’	0.65±0.13
all residues, all heavy atoms	1.05±0.10
PROCHECK [38] G-factors raw score	
(φ and ψ/all dihedral angles)^c^	0.34/0.22
PROCHECK [38] G-factor Z - score	
(φ and ψ/all dihedral angles)^c^	1.65/1.30
MOLPROBITY[39] clash score (raw/Z - score)^c^	20.88/−2.06
AutoQF R/P/F/DP scores [40] (%)	96/97/96/81
Ramachandran plot summary^c^	
most favorable regions	92.7
additionally allowed regions	7.3
generously allowed regions	0.0
disallowed regions	0.0

a Related to pairs with non-degenerate chemical shift.

b Regular secondary element: α-helical residues 173–191, 204–235, 240–253, 262–280, 284–301, 303–308 and 311–319.

c Ordered residues: 172–192,194–198, 204–235, 238–255, 262–321 with dihedral angle order parameters S(φ) and S(ψ) > 0.90. Z-scores were computed relative to corresponding structure quality measures for high resolution X-ray crystal structures [42].

**Figure 3:**
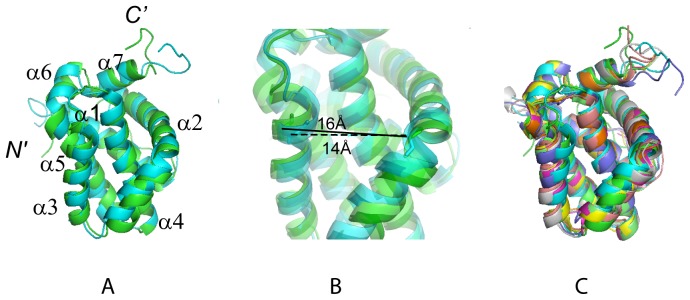
Superposition of selected Mcl-1 structures. (A) Structures of hMcl-1(171–327) (green) and mMcl-1(152–308) (cyan, PDB accession code 1wsx) after superposition of the backbone N, C^α^ and C’ atoms of the α-helices for minimal rmsd. (B) Ribbon drawing (zoomed into (A)) showing the different binding groove widths of human (green) and mouse (cyan) protein. The distances between the Cα-atoms of residues His 224 in helix α2 (His 205 in mMcl-1) and His 252 (His 233 in mMcl-1) at the C-terminus of helix α3 are highlighted: ∼16 Å in hMcl-1(171–327) and ∼14 Å mMcl-1(152–308) (C) Superposition as in (A) of hMcl-1(171–327) (green) and mMcl-1(152–308) (cyan, 1wsx), and six selected Mcl-1 complex structures (see also Table 2): human Mcl-1 complexed with Bim BH3 (magenta, 2nla); chimeric rat-human rMcl-1(171–208)hMcl-1(209–327) complexed with mouse mNoxaB BH3 (yellow, 2rod); mouse mMcl-1(152–308) complexed with mouse NoxaA BH3 (pink, 2roc); mouse mMcl-1(152–308) complexed with mouse Puma BH3 (grey, 2jm6); mouse mMcl-1(152–308) complexed with mouse NoxaB BH3 (purple, 2rod); chimeric rat-human mMcl-1(171–208)hMcl-1(209–327) complexed with human Bim BH3 (orange, 2nl9); chimeric rat-human mMcl-1(171–208)hMcl-1(209–327) complexed with human Bim BH3(L62A, F68A) (light green, 3d7v).The figures were prepared with the programs MOLMOL [36] and PYMOL [37].

Including our hMcl-1(171–327) structure, twenty atomic resolution structures containing different Mcl-1 constructs are currently deposited in the PDB. In addition to the two ‘apo’ proteins hMcl-1(171–327) and mouse mMcl-1(152–308) [10] [PDB accession code 1wsx, 89% sequence identity with the human protein], the structures for nineteen protein-ligand complexes were deposited (Table 2) [9],[11]–[18]. Clearly, the large number of available structures reflects the outstanding interest in Mcl-1 as a target for the development of new cancer drugs. Superposition of the α-helices reveals, as expected, close structural similarity for all Mcl-1 proteins structures (Figure 3): the root mean square deviation (rmsd) values range from 1.05 to 1.54 Å relative to hMcl1-1(171–327) (Table 2). However, comparison of the two apo protein structures of hMcl-1(171–327) and mMcl-1(152–308) with the complex structures shows that the binding pocket is widened upon complex formation (Table 2): the distances between the C^α^-atoms of residues His 224 in helix α2 (His 205 in mMcl-1) and His 252 (His 233 in mMcl-1) at the C-terminus of helix α3 are, respectively, ∼16 Å and ∼14 Å in hMcl-1(171–327) and mMcl-1(152–308), and ∼18–21 Å in the complexes.

**Table 2:** Rmsd values for comparison of the NMR structure of hMcl-1(171–327) with the structures of mouse mMcl-1(152–308) and Mcl-1complexes.^a^

Mcl-1 structures	172–193, 203–321^b^	α1–α7^c^	209–321^b^	α2–α4^d^	α2–α7^d^	dCA_224,252_ d
mMcl-1^e^	1.60±0.09	1.52±0.06	1.61±0.10	1.21±0.07	1.53±0.06	13.2–14.6
hMcl-1-hBim^f^	1.76±0.10	1.41±0.06	1.87±0.11	1.52±0.09	1.53±0.09	19.9
rMcl-1-hMcl-1-mNoxaB^g^	1.46±0.12	1.05±0.08	1.53±0.14	1.00±0.10	1.08±0.09	19.9
mMcl-1-mNoxaA h	1.52±0.11	1.16±0.07	1.59±0.13	1.11±0.11	1.18±0.08	18.8–20.2
mMcl-1-mPuma^i^	1.57±0.09	1.30±0.08	1.59±0.11	1.24±0.11	1.30±0.09	18.3–19.9
mMcl-1-mNoxaB^j^	1.46±0.09	1.13±0.05	1.53±0.11	1.12±0.08	1.18±0.06	18.3–19.6
rMcl-1-hMcl-1-hBim^k^	1.75±0.09	1.44±0.06	1.86±0.11	1.58±0.09	1.56±0.07	19.9
rMcl-1-hMcl-1-hBim(L62A, F78A) l	1.80±0.09	1.44±0.06	1.90±0.11	1.53±0.08	1.55±0.07	19.8
hMcl1-hBidBH3^J^	1.84±0.15	1.54±0.09	1.57±0.20	1.30±0.15	1.30±0.12	20.8–21.4
hMcl1-hBIMBH3^k^	1.38±0.16	1.06±0.07	1.43±0.18	0.93±0.10	1.09±0.09	19.4
hMcl1-BimL12Y^l^	1.75±0.13	1.50±0.08	1.85±0.15	1.48±0.12	1.59±0.09	20.2
hMcl1-BimBH3 2dA^m^	1.75±0.13	1.50±0.08	1.84±0.14	1.46±0.12	1.57±0.09	19.7
hMcl1-BimBH3 F4aE^n^	1.75±0.13	1.47±0.08	1.84±0.15	1.44±0.08	1.55±0.08	19.9
hMcl1 -B7^o^	1.73±0.14	1.46±0.08	1.83±0.11	1.42±0.11	1.54±0.08	19.6–19.9
hMcl1–hMcl1BH3^p^	1.47±0.16	1.21±0.09	1.48±0.18	1.08±0.15	1.24±0.11	20.3
hMcl1-BaxBH3^q^	1.69±0.12	1.46±0.08	1.79±0.13	1.43±0.12	1.54±0.09	19.4
mMcl1-NoxaBH3^r^	1.43±0.16	1.20±0.10	1.48±0.17	1.10±0.13	1.23±0.11	19.1
hMcl1-compound53^s^	1.45±0.12	1.22±0.08	1.48±0.14	1.19±0.10	1.28±0.08	18.7
hMcl1-compound60^t^	1.45±0.13	1.22±0.08	1.47±0.12	1.12±0.09	1.25±0.08	17.9–19.6
hMcl1-BH3^u^	1.51±0.16	1.22±0.09	1.57±0.18	1.06±0.12	1.27±0.10	20.3

a Average pairwise rmsd values (Å) were calculated for backbone heavy atoms N, C^α^, and C’ between the 20 conformers of Mcl-1(171–327) and corresponding polypeptide segments in the other structures. The distances dCA (in Å) between the C^α^-atoms of residues His 224 in helix α2 (His 205 in mMcl-1) and His 252 (His 233 in mMcl-1) at the C-terminus of helix α3 are provided as a measure for the width of the BH3 binding groove.

b Residue numbers are for hMcl-1(171–327); residues 194–202 were excluded since one structure (2nl9^k^) does not contain the corresponding residues; residues 172–193 and 203–321 correspond to residues 153–174 and 184–302 in mMcl-1, and residues 209–321 correspond to residues 190–302 in mMcl-1.

c Helices α1–α7 in hMcl-1 comprise residues 173–191, 204–235, 240–253, 262–280, 284–301, 303–308 and 311–319; the corresponding residues in mMcl-1 are: 155–172, 185–216, 221–234, 243–261, 265–282, 284–289 and 292–300.

d Helices α2–α7 in hMcl-1 and residues 204–208 (numbers in hMcl-1) were excluded

e Mouse mMcl-1(152–308), PDB accession code 1wsx (the mean NMR coordinates were used) [10].

f Human hMcl-1 complexed with human hBim BH3, 2pqk [11].

g Chimiric rat-human rMcl-1(171–208)hMcl-1(209–327) complexed with mouse mNoxaB BH3, 2nla [9].

h Mouse mMcl-1 complexed with mouse mNoxaA BH3, 2rod [12].

i Mouse mMcl-1 complexed with mouse mPuma BH3, 2roc [12].

j Mouse mMcl-1 complexed with mouse mNoxaB BH3, 2jm6 [9].

k Chimiric rat-human Mcl-1 complexed with human hBim BH3, 2nl9 [9].

l Chimiric rat-human Mcl-1 complexed with human hBim (L62A, F68A), 3d7v [13].

J Human hMcl1 complexed with human Bid BH3, 2kbw [15].

k Human hMcl-1 complexed with human Bim BH3 mutant I2dY, 3kj0 [11].

l Human hMcl-1 complexed with human BimL12Y, 3io9 [16].

m Human hMcl1 complexed with human Bim BH3 mutant I2dA, 3kj1 [11].

n Human hMcl1 complexed with human Bim BH3 mutant F4aE, 3kj2 [11].

o Human hMcl-1 complexed with Mcl1 specific selected peptide B7, 3kz0 [41].

p Human hMcl-1 complexed with human Mcl1 BH3, 3mk8 [17].

q Human hMcl1 complexed with human Bax BH3, 3pk1.

r Mouse mMcl-1 complexed with mouse Noxa BH3, 4g35 [18].

s Human hMcl-1 complexed with 6-chloro-3-[3-(4-chloro-3,5-dimethylphenoxy)propyl]-1H-indole-2-carboxylic acid, 4hw2 [14].

t Human hMcl-1 complexed with 6-chloro-3-[3-(4-chloro-3,5-dimethylphenoxy)propyl]-1H-indole-2-carboxylic acid, 4hw3 [14].

u Human hMcl-1 complexed with human Mcl1 BH3, 4hw4 [14].

The fact that the human apo protein exhibits a somewhat wider binding groove than the mouse homologue (Table 2) can be, at least partially, ascribed to the side chain of Leu 246 in the human protein which is not buried as deeply as the corresponding Phe side chain in the mouse protein. Furthermore, when comparing the human and the mouse protein, differences are observed for the charge distributions in the BH3-binding groove (Figure 2): the human protein is negatively charged on the side of helix α3, while the corresponding surface of mouse protein is positively charged. This difference arises from Ser 255 corresponding to Lys 236 in the mouse protein. Remarkably, hMcl-1(171–327) is structurally more similar to the hMcl1(171–327)-hBim BH3 complex (Figure 3) than to apo mMcl-1(152–308) (Table 2).

Taken together, structural comparisons show that, in spite of the 89% sequence identity between human and mouse protein, the availability of the human hMcl-1(171–327) structure can be expected to be of critical importance for supporting future design of cancer drugs.

## Materials and Methods

### NMR Sample Preparation

Preliminary studies showed that hMcl-1(171–327) (UniProtKB/Swiss-Prot ID Q07820/MCL1_HUMAN) is not stable in solution. However, the mutant Cys 286 → Ser is stable for several weeks at concentrations ∼0.7 mM, and both wild-type and mutant bind the Bim-BH3 peptide with the same affinity (K_d_ ∼ 60 pM) in a Biacore assay. Hence, we solved the NMR structure of hMcl-1(171–327) Cys 286 → Ser referred to as hMcl-1(171–327) in this publication.

hMcl-1(171–327) was cloned, expressed, refolded and purified following standard protocols to produce a uniformly ^13^C, ^15^N-labeled protein sample [19]. Briefly, the gene was cloned into a pET21d (Novagen) derivative yielding plasmid pSR482-21.1. The resulting construct contains seven nonnative residues at the C-terminus (LHHHHHH) to facilitate protein purification. *Escherichia Coli* BL21 (DE3) pMGK cells, a codon enhanced strain, were transformed with pMcl1-21.1, and cultured in MJ9 minimal medium [20] containing (^15^NH_4_)_2_SO_4_ and *U*-^13^C-glucose as sole nitrogen and carbon sources. Double-washed inclusion bodies containing hMcl-1(171–327) were solubilized in 8 M buffered guanidine hydrochloride (VWR) containing 5 mM DTT, slowly diluted into nine volumes of 20 mM Tris-HCl, 250 mM NaCl, 0.5 M urea, 10% glycerol, pH 7.4, and refolded within 72 hours at 4°C. hMcl-1(171–327) was purified using a Talon affinity resin (Clontech) applied to a HiTrap SP High Performance column (GE Healthcare). The final yield of purified *U*-^13^C, ^15^N protein (> 98% homogeneous by SDS-PAGE; 20.3 kDa by MALDI-TOF mass spectrometry) was ∼25 mg/L. In addition, a *U*-^15^N and 5% biosynthetically directed fractionally ^13^C-labeled sample [21] was generated for stereo-specific assignment of isopropyl methyl groups. NMR samples were prepared at 0.7 mM protein concentration. An isotropic overall rotational correlation time of ∼10 ns was inferred from ^15^N spin relaxation times indicating that hMcl-1(171–327) is monomeric in solution.

### NMR spectroscopy

NMR spectra were recorded at 25°C. Five G-matrix Fourier transform (GFT) NMR experiments [22],[23] and a simultaneous 3D ^15^N/^13^C^aliphatic^/^13^C^aromatic^-resolved NOESY [24],[25] spectrum (mixing time 60 ms; measurement time: 48 hours) were acquired on a Varian INOVA 750 MHz spectrometer equipped with a conventional probe. 2D constant-time [^13^C,^1^H]-HSQC spectra (18 hours) were recorded for the 5% biosynthetically directed fractionally ^13^C-labeled sample on a Varian INOVA 600 MHz spectrometer equipped with a cryogenic probe as was described [21],[26]. Spectra were processed and analyzed using the programs NMRPipe [27] and XEASY [28].

Sequence specific backbone (H^N^, H^α^, N, C^α^) and H^β^/C^β^ resonance assignments were obtained by using (4,3)D HNNC ^αβ^ C ^α^ (63 hours)/(4,3)D C ^αβ^ C ^α^(CO)NHN (62 hours), and (4,3)D H ^αβ^ C ^αβ^ (CO)NHN (69 hours) [23] along with the program AUTOASSIGN [29]. More peripheral side chain chemical shifts were assigned with aliphatic (4,3)D HCCH (87 hours) [23] and 3D ^15^N/^13^C^aliphatic^/^13^C^aromatic^-resolved [^1^H,^1^H]-NOESY [24],[25]. Overall, assignments were obtained for 96% of backbone and ^1^H^β^/^13^C^β^ resonances and for 93% of the side chain resonances which are assignable with the NMR experiments listed above (excluding the N-terminal NH_3_^+^, Pro ^15^N, ^13^C’ preceding prolyl residues, Lys NH_3_^+^, Arg NH_2_, OH, side chain ^13^C’ and aromatic ^13^C^γ^). Furthermore, 100%/100% of Val and Leu isopropyl moieties with non-degenerate proton chemical shifts were stereo-specifically assigned (Table 1). Chemical shifts were deposited in the BioMagResBank [30] (accession code 19654). ^1^H–^1^H upper distance limit constraints for structure calculations were obtained from NOESY (Table 1). In addition, backbone dihedral angle constraints were derived from chemical shifts using the program TALOS [31] for residues located in well-defined secondary structure elements (Table 1). The programs CYANA [32],[33] and AUTOSTRUCTURE [34] were used in parallel to assign long-range NOEs [24]. The final structure calculations were performed using CYANA followed by explicit water bath refinement using the program CNS [35].
